# An Innovative Porous Nanocomposite Material for the Removal of Phenolic Compounds from Aqueous Solutions

**DOI:** 10.3390/nano8050334

**Published:** 2018-05-16

**Authors:** Antonio Turco, Anna Grazia Monteduro, Elisabetta Mazzotta, Giuseppe Maruccio, Cosimino Malitesta

**Affiliations:** 1 Dipartimento di Scienze e Tecnologie Biologiche ed Ambientali (Di.S.Te.B.A.), Università del Salento, via Monteroni, 73100 Lecce, Italy; elisabetta.mazzotta@unisalento.it (E.M.); cosimino.malitesta@unisalento.it (C.M.); 2 CNR NANOTEC—Institute of Nanotechnology c/o Campus Ecotekne, Via Monteroni, 73100 Lecce, Italy; annagrazia.monteduro@unisalento.it (A.G.M.); giuseppe.maruccio@unisalento.it (G.M.); 3 Dipartimento di Matematica e Fisica, Università del Salento, Via per Arnesano, 73100 Lecce, Italy; 4 National Institute of Gastroenterology “S. De Bellis” Research Hospital, via Turi 27, 70013 Castellana Grotte, Bari, Italy

**Keywords:** carbon nanotubes, phenol, phenolic compound, remediation, oil mill wastewater

## Abstract

Energy efficient, low-cost, user-friendly, and green methods for the removal of toxic phenolic compounds from aqueous solution are necessary for waste treatment in industrial applications. Herein we present an interesting approach for the utilization of oxidized carbon nanotubes (CNTs) in the removal of phenolic compounds from aqueous solution. Dried pristine CNTs were stably incorporated in a solid porous support of polydimethylsiloxane (PDMS) facilitating the handling during both oxidation process of the nanomaterial and uptake of phenolic compounds, and enabling their safe disposal, avoiding expensive post-treatment processes. The adsorption studies indicated that the materials can efficiently remove phenolic compounds from water with different affinities towards different phenolic compounds. Furthermore, the adsorption kinetics and isotherms were studied in detail. The experimental data of adsorption fitted well with Langmuir and Freundlich isotherms, and pseudo-second-order kinetics, and the results indicated that the adsorption process was controlled by a two-step intraparticle diffusion model. The incorporation of CNTs in polymeric matrices did not affect their functionality in phenol uptake. The material was also successfully used for the removal of phenolic compounds from agricultural waste, suggesting its possible application in the treatment of wastewater. Moreover, the surface of the material could be regenerated, decreasing treatment costs.

## 1. Introduction

Petrochemical, pharmaceutical, steel, and agricultural industries produce a huge number of phenol and phenolic compounds, which are common contaminants in industrial wastes [1]. Due to their toxicity and carcinogenicity, many of these kinds of compounds have been classified as hazardous pollutants [2]. Exposure to phenolic compounds, even at low concentrations and short-term exposure, leads to high irritation to eyes, skin, and mucous, and causes headache and dizziness. Long-term exposure results in high blood pressure and severe liver and kidney damage.

Different technologies, like electrochemical oxidation [3,4,5], (electro)chemical coagulation [6,7,8], solvent extraction [9,10], bioremediation [11,12,13], and photocatalytic degradation [14,15,16], have been developed in the years to remove phenolic compounds from wastewater, however, all these methods require high energy input and/or additional chemicals, making the treatment expensive. Moreover, secondary pollutants are often generated during the treatment process. Thus, there is a need to develop energy efficient and low-cost methods to separate/remove phenolic compounds from aqueous wastes. In this view, adsorption techniques can offer an attractive alternative. Several adsorbents, such as nanoparticles [17,18], activated carbon [19,20,21], resins [22,23,24], and other low-cost adsorbents [25], have been used for removal of phenolic compounds. Carbon nanotube (CNT) materials have demonstrated good potential for adsorption of phenols from aqueous solutions [26,27,28]. Their large surface area, porosity, and functional groups are features that can enhance their adsorption efficiency [29]. However, although these materials showed good affinity towards phenolic compounds, the major limit in their application is that collection of the carbon nanotube powder after the remediation process is cumbersome, and require post-processes, such as filtration with membrane with nanometer size pores and/or centrifugation, thus limiting their real and large-scale application. Fixing the CNT powder in an easy way in solid compact without compromising the chemical affinity to phenols could be an interesting solution for the usability in the treatment of wastewater. Herein, we report an approach to utilizing carbon nanotubes immobilized on a porous polydimethylsiloxane (PDMS) sponge for the removal of phenolic compounds. The PDMS acts as a support for CNTs and its microporous structure permits it to have a high amount of nanomaterial on the sponge surface in a reduced volume. The as-prepared material can remove different phenolic compounds with different affinities, depending on the type and number of functional groups present as substituents on the aromatic benzenic ring of phenols.

The adsorption mechanisms and kinetics were studied through different theoretical models. The system was demonstrated to be effective in the purification of complex matrices, such as oil mill wastewater, an agricultural waste obtained after oil production. The composite foams have good performance, but most importantly, they can be easily handled and disposed. The material recyclability is also demonstrated, making the system an effective and low-cost method for phenol adsorption from aqueous wastewater.

## 2. Materials and Methods

### 2.1. Materials

The PDMS Prepolymer Kit (Sylgard 184), comprising monomer and curing agent, were purchased from Dow Corning, Midland, MI, USA. Multiwalled carbon nanotubes (MWNTs) with a diameter of 25.4 ± 4 nm (see Figure S1) were provided by Nanostructured and Amorphous Materials, Inc., Los Alamos, NM, USA. All the other reagents were purchased from VWR International srl, Milano, Italy, and used as received. Olive mill wastewater was collected from a three phase continuous extraction unit, Miggiano, Lecce, southern Italy. All the solutions were prepared with ultrapure water (Millipore Milli-Q, 18.2 MΩ cm^−1^).

### 2.2. Fabrication of PDMS/MWNT Porous Nanocomposite

Porous PDMS/MWNT nanocomposite was prepared according to a modified procedure previously developed by our group [30]. Briefly, glucose particles were mixed for 30 min with MWNTs at 3% *w*/*w* under dry conditions at room temperature, in a propylene centrifuge tube, thus allowing the adsorption of carbon nanotubes on glucose microparticle surface without the need for other reagents. In particular, as previously reported, sugar crystals could mechanically break the π–π stacking between the MWNTs favoring the disruption of the bundles, and the adsorption of the nanomaterials on glucose crystal surfaces [30]. On the top of the mixture, an appropriate amount of PDMS prepolymer and curing agent in the ratio of 10:1 diluted at 40% in wt, in hexane, was added. The mixture was then centrifuged at 8000 rpm for 20 min to pack glucose crystals and promote the infiltration of the prepolymerization mixture among the sugar templates. The as-obtained composites were then cured at 60 °C overnight to accomplish polymerization. The cured sample was removed from the centrifuge tube and soaked in boiling water to dissolve glucose microparticles, and then sonicated with warm water and ethanol to remove residual template microparticles, and not entrapped MWNTs.

### 2.3. Oxidation of PDMS/MWNT Porous Nanocomposite

As-prepared PDMS/MWNT porous nanocomposite was placed in water under reduced pressure to saturate all the cavities of the material with water. Then, concentrated HNO_3_ was added drop by drop to the solution, until a concentration of 3 M was reached. The reaction was kept at room temperature for 2 h. The PDMS/MWNT sponge was removed from the solution and washed repetitively with water until the pH of the washing solution become neutral, and dried under vacuum. After that, the nanocomposite material was dipped in H_2_O_2_ 30% *w/w* for 2 h, and then washed three times with water. The obtained black spongeous material (PDMS/MWNT_ox_) was dried overnight at 60 °C.

### 2.4. Characterization Techniques

The morphological characterization of PDMS and PDMS-CNT sponge samples was performed using a scanning electron microscope (SEM, Carl Zeiss Merlin, Jena, Germany) in top view configuration, and applying an acceleration voltage of 3 kV for PDMS sponges and 5 kV for PDMS-CNT samples. Secondary electron and in-lens detectors were employed for low and high magnification, respectively. Morphological characterization of as-received MWNTs was performed using a transmission electron microscope (TEM, Philips EM208, Philips Scientifics, Eindhoven, The Netherlands) applying an acceleration voltage of 100 kV.

XPS measurements were performed in triplicate with an AXIS ULTRA DLD (Kratos Analytical, Manchester, UK) electron spectrometer using a mono-Al Kα source (1486.6 eV) operating at 225 W (15 kV, 15 mA). Survey scan spectra were recorded using a pass energy of 160 eV and a 1 eV step. The hybrid lens mode was used for all measurements. In each case, the area of analysis was about 700 μm × 300 μm. The base pressure in the analysis chamber was 2 × 10^−9^ Torr. During the acquisitions, a system of neutralization of the charge was used. The binding energy (BE) scale was referenced to the C 1s line of alkylic carbon (285 eV). Processing of the spectra was accomplished with CasaXPS Release 2.3.16 software (Casa Software Ltd., Teignmouth, UK).

### 2.5. Phenol Determination

Quantitative determination of the concentration of the phenolic compounds was performed by UV-vis spectrophotometry using a Cary UV 50 spectrophotometer. The calibration curves (absorbance vs. concentration) for each tested phenolic compound were obtained at their maximum absorption wavelength. Since analytical wavelength and sensitivity for phenolic compounds are affected by the solution pH, calibration curves were obtained at the same pH of the adsorption experiments (pH adjusted with NaOH or HCl).

### 2.6. Adsorption Experiments

For the adsorption studies, 5 mL of aqueous solution at pH = 10 with phenolic compounds were prepared at different concentrations, which are reported in the different results and discussion sections. Then, a proper amount of the PDMS/MWNT_ox_ nanocomposite was immersed in the solution and shacked at room temperature for 24 h. Small aliquots were collected from the solution at specific time intervals, namely 0.5, 1, 1.5, 2, 2.5, 3, 4, 20, and 24 h (if not otherwise indicated), for further spectrophotometric analysis. The time dependent removal efficiency and the equilibrium adsorption capacity of the sponges were calculated by Equations (1) and (2), respectively:

(1) $$\begin{array}{ccc} removal & efficiency & \left(\%\right) \end{array}=\left[\frac{\left(C_{0}-C_{t}\right)}{C_{0}}\right]\times 100$$

(2) $$\begin{array}{ccc} adsorption & capacity, & q_{e}\left(\frac{mg}{g}\right) \end{array}=\left[\frac{\left(C_{0}-C_{e}\right)}{W}\right]\times V$$

Here, the following abbreviations were applied: *C*_0_ is the initial concentration of phenolic compounds in ppm (mg/Kg), *C_t_* is the concentration expressed in ppm at a given time *t* (hours), *q_e_* is the adsorption capacity (mg/g) at the equilibrium, *C_e_* is the phenolic compound concentration expressed in ppm at the equilibrium conditions (usually after 20 h), *V* is the volume of the solution in liters, and *W* is the weight of the sponge in grams.

### 2.7. Removal of Phenolic Compound from Olive Mill Wastewater (OMW)

OMW were obtained from a three-phase continuous extraction unit from Miggiano, Lecce (southern Italy). The OMW samples were first stored in a polyethylene tube at ambient temperature. PDMS/MWNT_ox_ (1.2% in weight) were added to OMW and shacked with a rotary shaker, and aliquots were collected from the solution after 24 h for further spectrophotometric analysis. Concentration of phenols in OMW was determined by the colorimetric reaction with Folin-Ciocalteu reagent. Briefly 50 µL of the OMW was mixed with 250 µL of Folin-Ciocalteu reagent, 240 µL of water, and 2.5 mL of a solution of NaHCO_3_ (7% *w/w*), and the mixture was shaken for 90 min in dark. The blue color formed was measured at 760 nm. The phenolic compound concentration of OMW samples, as determined by the Folin-Ciocalteu method, were reported as caffeic acid equivalents by reference to a standard curve [31].

## 3. Results

### 3.1. Preparation and Characterization of PDMS/MWNTs_ox_ Spongeous Material

**Scheme 1:**
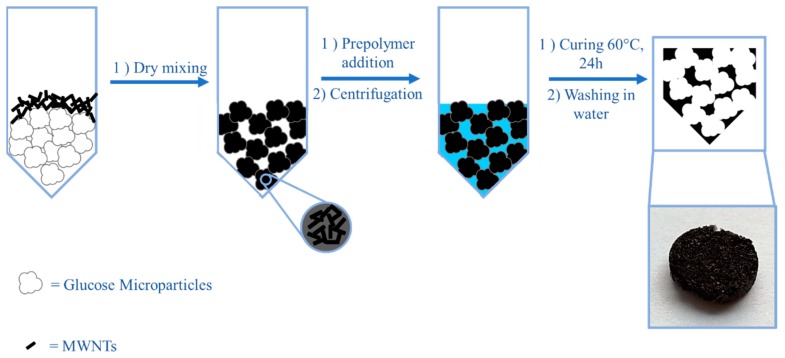
Schematic representation of the preparation of the polydimethylsiloxane (PDMS)-multiwalled carbon nanotube (MWNT) sponge, and photograph of the as-obtained material.

Porous PDMS/MWNT sponges were fabricated by polymerization of the prepolymer and the curing agent in hexane in the presence of glucose microparticles covered with MWNTs. The color of the as-obtained materials was black, indicating the presence of MWNTs as filler in the as prepared sponge (Scheme 1).

The as-prepared sponges were then chemically oxidized with a mild treatment based on nitric acid, followed by hydrogen peroxide to increase the number of –OH, –C=O, and –COOH groups on the MWNT surface, minimizing the nanotube damage [32]. The oxidation process, including post-treatment procedure (see Section 2) were facilitated by the fact that MWNTs are entrapped in a macroscopic polymeric structure, thus speeding-up the procedure, avoiding time expensive steps, such as filtration and/or centrifugation.

**Figure 1:**
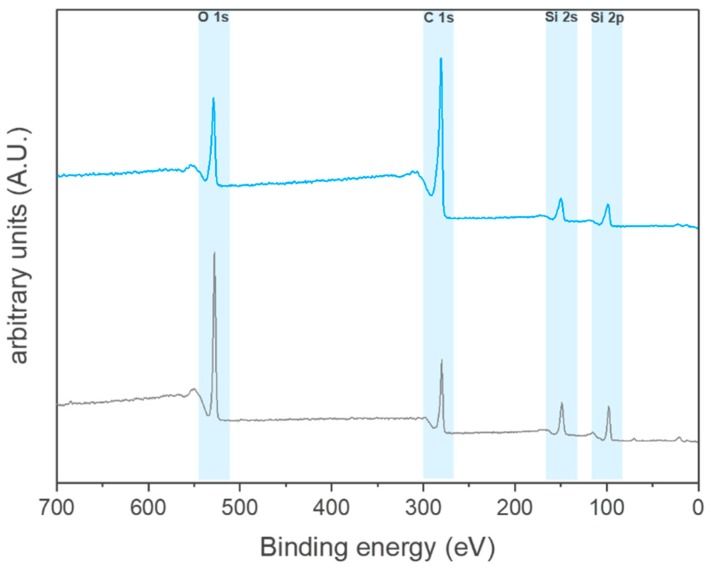
XPS survey spectra of PDMS/MWNT sponge before (blue curve) and after (grey curve) mild oxidation treatment.

The effective functionalization of MWNTs was verified by XPS. The survey scan spectra in Figure 1 reveal the chemical composition of the uppermost surface of the PDMS/MWNT spongeous material before (blue curve) and after (grey curve) the mild oxidation treatment. The major peaks observed in the spectra were due to C, O, and Si photoelectron peaks. The O 1s/C 1s ratio before the mild acidic treatment was equal to 0.57 ± 0.01. After the oxidation process, the peak relative to O 1s photoelectron became more prominent, and O 1s/C 1s ratio increased to 2.11 ± 0.31, indicating that oxygen-containing moieties were present on the surface of the nanocomposite material [33]. Moreover, iron photoelectron peaks are not evident on both samples, thus suggesting the low metallic content of MWNTs, as also clearly visible in the TEM image of the as-received MWNTs (Figure S1).

**Figure 2:**
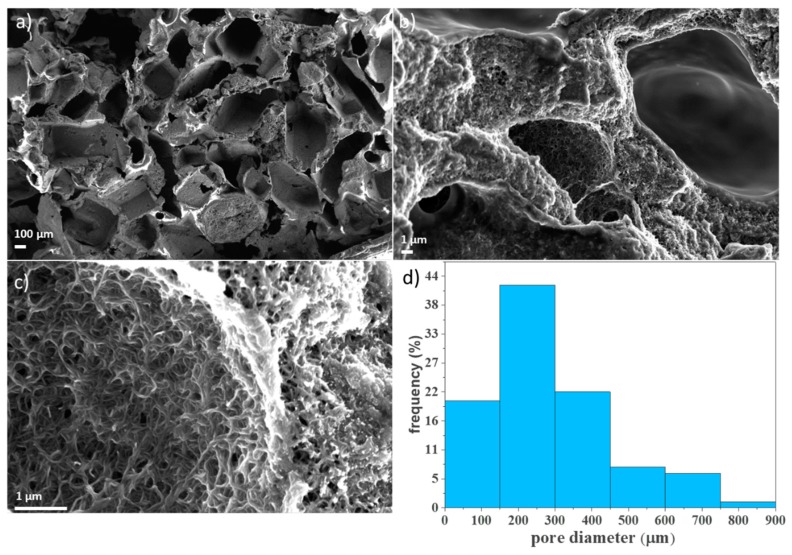
SEM images of porous PDMS/MWNTox sponge at different magnification (**a**–**c**). In (**d**) pore size distribution is reported.

SEM images (Figure 2a–c) of PDMS/MWNT_ox_ sponge cross-sections evidence an interconnected 3D framework. Figure 2d shows the pore size distribution, which has an average pore dimension of 290 ± 170 µm. At higher magnification, the sponges exhibit the presence of the nanomaterial close to the surface of the pores (Figure 2c) with oxidized MWNTs homogenously dispersed inside the polymeric matrices with random orientation.

The amount of MWNT_ox_ per gram of sponge were estimated to be 68.7 mg, by comparing the density of PDMS and PDMS/MWNT_ox_ sponges. Moreover, PDMS/MWNT_ox_ evidenced conductive behavior if contacted with a multimeter (Figure S2), thus confirming the presence of a continuous carbonaceous network, the partial integrity of aromatic continuity in MWNTs_ox_ inside polymeric matrices, and the presence of MWNTs_ox_ on the top layer of the sponge, thus suggesting the possibility of the nanomaterial to interact with molecules in a solution in contact with the surface of the nanocomposite.

### 3.2. Adsorption of Phenolic Compounds to PDMS/MWNT_ox_ Porous Nanocomposite

**Figure 3:**
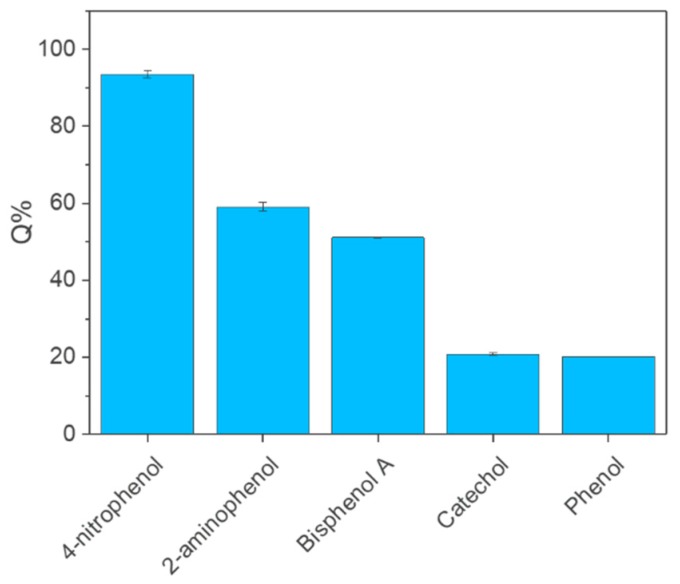
Removal efficiency (Q%) of different phenols for PDMS/MWNT_ox_ sponge.

The PDMS/MWNT_ox_ nanocomposite were dipped in water solutions containing different phenolic compounds at the same concentration for 24 h, and the removal efficiency was calculated as described in the Section 2. Results are summarized in Figure 3. It is important to note that during the process, neither desorption of MWNTs from the polymeric matrices nor damage of the material were observed by naked eye. Although all the phenolic compounds were at the same concentration (i.e., 0.18 mM), the removal efficiency (Q%) is significantly different for each phenolic compound, suggesting different affinities of the spongeous materials towards the different phenolic compounds. The phenolic compounds used in this study can be classified into two groups: (1) nitroaromatic compound (i.e., 4-nitrophenol), (2) polar aromatic phenolic compounds (i.e., phenol, catechol, bisphenol-A, and 2-aminophenol). Nitroaromatic phenolic compound adsorbed more strongly than polar aromatic compounds, and adsorption of polar aromatic phenolic compounds is influenced by the number and the type of functional groups. For example, adsorption of catechol is slightly higher than phenol and 2-aminophenol adsorbed more than catechol. These results are in agreement with the adsorption behavior of different phenolic compounds on MWNTs previously observed [27]. The different removal efficiencies recorded on PDMS/MWNT_ox_ sponges depending on the type and the number of functional groups could suggest different interaction mechanisms between phenols and the nanocomposite.

Nitroaromatic compounds are highly polar, and act as strong electron acceptors when interacting with adsorbents containing structures with high electron polarizability. The other tested phenolic compounds are highly polar but non-electron acceptors. It appears that the higher adsorption of 4-nitrophenol (4-NP) was due to π–π electron donor-acceptor interactions between the nitroaromatic (π-acceptors) and the surface of PDMS/MWNT_ox_ with regard to graphitic surface (π donors) of carbon nanotubes. The π–π electron donor-acceptor is a specific, non-covalent interaction that can be established between electron-rich and electron poor moieties. Donor strength increases with the electron-donating ability of substitutes or π system polarizability. Acceptor strength is dependent on electron-withdrawing ability and the number of substitutes. The graphitic surface of MWNTs is highly polarizable, and acts as an electron donor when in contact with acceptors, such as nitroaromatic compounds [28]. As an alternative adsorption mechanism, H bonding between nitro groups (H acceptor) and functional groups (e.g., –COOH, –CO, –CHO) on the MWNT_ox_ surface inside the sponges might also be considered. In this case, the removal efficiency of 4-nitrophenol would increase with decreasing pH, due to the transition of oxidized multiwalled carbon nanotube moieties, such as –COO^−^ to the protonated form, –COOH, that can form H-bonds with nitro group of 4-NP. However, adsorption studies at different pH showed that when varying pH from 4 to 11 (Figure S3), the adsorption increases more than 30%, reaching a maximum at pH ~ 10, in which all carbon nanotubes functional groups are mostly in the deprotonated form. Therefore, π–π electrons-donor-acceptor interactions of nitrophenolic compounds with PDMS/MWNT_ox_ sponges could be the most plausible mechanism of adsorption.

Similarly, two adsorption mechanisms, i.e., π–π electron donor-acceptor interactions or H-bonding, can be hypothesized in the case of polar organic compounds. With the aim to evaluate the occurring adsorption mechanism, the pH effect on the sorption of phenol (Ph) by PDMS/MWNT_ox_ sponge was evaluated, with pH ranging from ~2 to ~11. Phenol was chosen because it is the aromatic phenolic compound with lower removal efficiency and simpler chemical composition. As evident (Figure S4), the removal efficiency increases with pH increase reaching the maximum at pH ~ 11. For higher pH, we observed a dramatic decrease in removal efficiency due to the increased electrostatic repulsion between the dissociated phenol (pKa ~ 10) and negatively charged PDMS/MWNT_ox_ surface at these pH values. The dissociation of –OH groups on phenol may inhibit the formation of hydrogen bonds between the polar aromatic compound and MWNT surface at higher pH [27]. However, the increase of the removal efficiency of phenol of about 20% at pH ~ 11 suggested the low significative involvement of hydrogen bonding between phenol and PDMS/MWNT_ox_ sponge. As already reported in literature, the increasing pH may alter the properties of polar aromatics, such as π-donating strength, thus increasing their adsorption affinities to the MWNT_ox_ surface [27]. However, the mechanism of this process is still unknown. Therefore, the adsorption mechanism for phenol could be also attributed to π–π electron donor-acceptor interaction. In more detail, hydroxyl is an electro-donating functional group which can increase the π donating strength of the host aromatic ring. The MWNTs_ox_ on the surface of PDMS sponge can act as electron acceptors, favoring adsorption of phenol on the surface with an opposite behavior with respect to that observed for nitroaromatic compound adsorption. It is well known, in fact, that the graphene surface of MWNTs could be highly polarizable, and may act as an amphoteric adsorbent attracting π-acceptor to the electron-rich graphene surface area near edges, and π-donors to the central regions, being relatively electron poor [27].

The proposed mechanism can be easily translated to the increase in removal efficiency for catechol due to the presence of two hydroxyl groups that can increase donor strength of aromatic benzene. Moreover, the removal efficiency could also increase if a –OH group of catechol is substituted with an amino group, due to the higher electron-donating effect of amino group with respect to hydroxyl [34]. The increased removal efficiency for bisphenol A, with respect to phenol and catechol, could be explained both by the π–π electron donor-acceptor interaction, and by the fact that larger molecules can twist themselves so that they match with the curvature surface, thus forming more stable complexes with the MWNT_ox_ surface [29].

**Figure 4:**
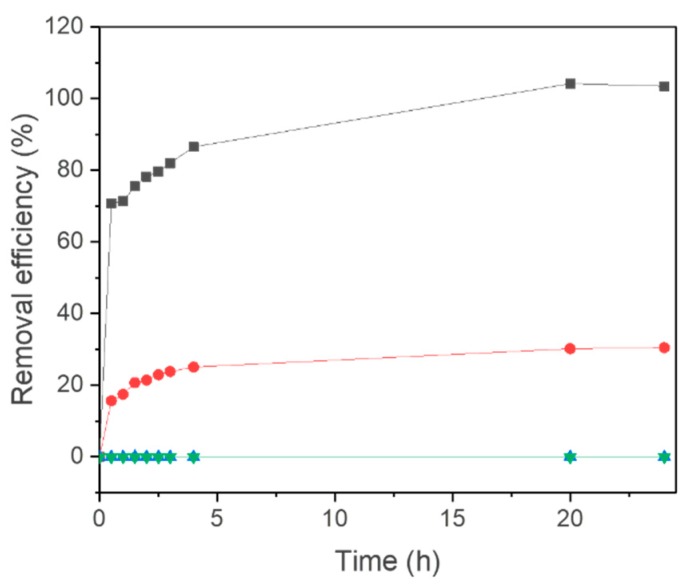
Time dependent removal efficiency (%) of 4-nitrophenol by the PDMS/MWNT_ox_ sponge (gray square), PDMS sponge (green square), and PDMS/MWNT sponge (blue triangle). The time dependent removal efficiency (%) of the phenol by the PDMS/MWNT_ox_ sponge is also reported (red circle).

To better characterize the adsorption properties of the nanocomposite material, the same amount of sponge was dipped in two different solutions containing the phenolic compounds, for which the material has demonstrated the highest and lowest removal efficiencies, namely 4-nitrophenol and phenol. As observable in Figure 4, in both cases the concentration of phenolic compounds decreases with time until a plateau is reached (e.g., 20 h). It is interesting to note that more than 90% of the total removal efficiency is reached after ~3.3 h for both the phenolic compounds, suggesting a relatively fast adsorption process. Figure 4 also reported adsorption kinetic studies for sponges made of PDMS (green square) and PDMS/MWNTs (blue triangle), for which the oxidation process was not performed before the exposure to 4-nitrophenol. In these cases, the uptake of nitroaromatic compound was not detectable, suggesting that PDMS acts only as a support for MWNT deposition, without affecting removal efficiency. Moreover, the oxidation of MWNTs on porous PDMS surface appears to be necessary for adsorption of 4-nitrophenol. It is well known in literature that for polar organic compounds, such as phenols, the adsorption tends to increase with increased CNT oxygen content, due to both enhanced electron donor-acceptor interactions and the depressed hydrophobic interaction [29]. Moreover, the low amount of oxygen moieties on entrapped MWNT surfaces before oxidation could avoid the diffusion of water inside polymeric matrices, due to the high hydrophobicity of both PDMS and MWNTs [34,35]. Similar results were also obtained in the case of phenol adsorption for a PDMS and PDMS/MWNT sponge.

**Figure 5:**
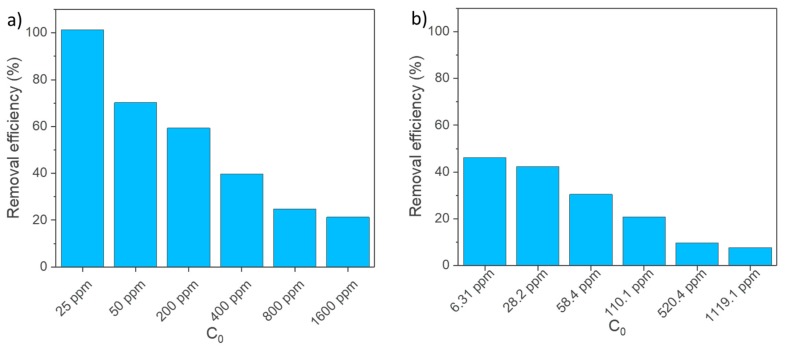
Effect of the initial 4-NP (**a**) and Ph (**b**) concentration on the removal efficiency (%).

Figure 5 reports the removal efficiency of 4-nitrophenol and phenol for different initial concentrations. As evident in both cases, as the initial phenolic concentration increases, the removal efficiency is reduced, indicating that the concentration of the species in solution is much higher compared to that of the phenolic compounds that can be adsorbed by the nanocomposite materials.

### 3.3. Adsorption Isotherms

Adsorption involves the accumulation of substances at an interface between the solid surface and the bathing solution. The equilibrium relationship between sorbent and sorbate are described by adsorption isotherms, that usually represent the ratio between the adsorbed quantity and that remaining in solution at a fixed temperature at equilibrium. In order to optimize the design of a sorption system, it is important to establish the most appropriate correlation for the equilibrium curve. An equilibrium is established when the amount of sorbate being adsorbed is equal to the amount being desorbed. Different isotherm equations are available in literature, and two of the most important isotherms, namely the Langmuir and Freundlich isotherms, are selected for this study.

The Langmuir equation is represented as follows:

(3) $$q_{e}=\frac{K_{L}q_{\max}C_{e}}{1+K_{L}C_{0}}$$

where *q_e_* (mg/g) and *C_e_* (g/L) are the amount of adsorbed phenolic compounds per unit weight of adsorbent and the unadsorbed phenolic compounds concentration in solution at equilibrium. *K_L_* (L/mg) is the affinity of the sorbate for binding sites, also defined as the Langmuir constant, and *q*_max_ (mg/g) is the maximum adsorption capacity when the surface is fully covered with phenolic compounds. The linearized form is represented as follows:

(4) $$\frac{C_{e}}{q_{e}}=\frac{1}{K_{L}q_{\max}}+\frac{C_{e}}{q_{\max}}$$

The plot of *C_e_*/*q_e_* against C_e_ should give a straight line with slope 1/*q*_max_ and intercept 1/*K_L_q*_max_.

**Figure 6:**
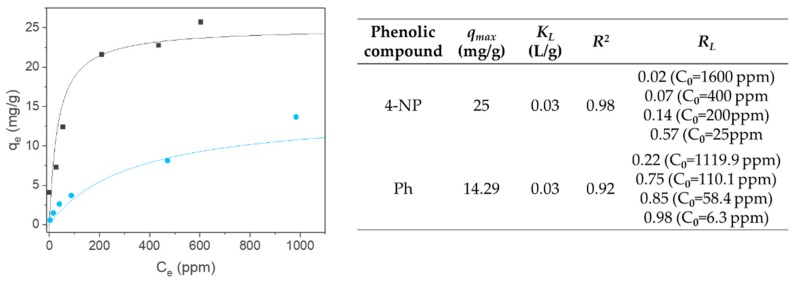
Fitting of experimental data with Langmuir isotherm model for 4-NP (black squares) and Ph (blue circles). The table reports calculated values from Equations (3)–(5).

The Langmuir isotherm is based on the assumption that the adsorbent is a two-dimensional array of energetically homogenous sites, in which only one molecule per site may be adsorbed, and interactions between any of the adsorbed molecules are also excluded. Consequently, the adsorption process has a monolayer nature. As evident in Figure 6 and table here reported, the Langmuir isotherm model fits the experimental data remarkably well for both the phenolic compounds tested (*R*^2^_4-NP_ = 0.98 and *R*^2^_Ph_ = 0.92).

From the fitting parameters, the parameters of the Langmuir model are calculated. A high *q*_max_ of 25 mg/g for 4-NP and 14.29 mg/g for Ph was found.

A dimensionless parameter *R_L_* was calculated for different concentrations of phenolic compounds. This parameter, called equilibrium parameter, is defined as

(5) $$R_{L}=\frac{1}{1+K_{L}C_{0}}$$

*R_L_* value indicates the type of isotherm to be irreversible (*R_L_* = 0), favorable (0 < *R_L_* < 1), linear (*R_L_* = 1, or unfavorable *R_L_* > 1) [36,37,38,39]. The results reported in the table at the bottom of Figure 6, revealed that the Langmuir adsorption is favorable for all the studied cases.

The adsorption of 4-NP and phenol on PDMS/MWNT_ox_ sponges were also studied with the Freundlich isotherm, which can be expressed as

(6) $$q_{e}=K_{F}C_{e}^{\frac{1}{n}}$$

where *K_F_* is the Freundlich constant that shows adsorption capacity of adsorbent, and *n* is a measure of intensity of adsorption. The equation can be expressed also by logarithmic form as

(7) $$\log q_{e}=\log K_{F}+\frac{1}{n}\log C_{e}$$

The plot of log *q*_e_ against log *C_e_* should give a straight line with slope 1/*n*.

**Figure 7:**
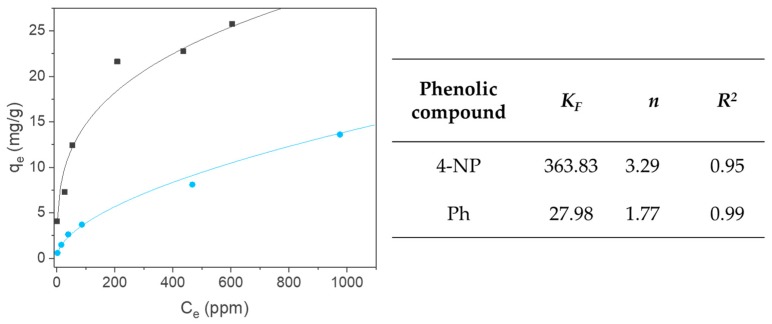
Fitting of experimental data with Freundlich isotherm model for 4-NP (black squares) and Ph (blue circles). The table reports calculated values from Equations (6) and (7).

The Freundlich isotherm assumed that the adsorption occurs on heterogenous sites with non-uniform distribution of energy level. The model describes reversible adsorption, and is not restricted to the formation of a monolayer. Also, this theoretical model fits the experimental data remarkably well for both the phenolic compounds, as clearly visible in Figure 7, and by *R*^2^ values reported in the table at the bottom of the figure. Similarly, as observed for Langmuir model, dimensionless parameter n shows a value between 1 and 10 in the whole tested concentration range, suggesting that the adsorption process of phenolic compounds is favorable for the tested concentrations [36,40].

As observed, both Langmuir and Freundlich models fit the experimental data, suggesting the possibility of monolayer phenolic compounds formation on MWNT_ox_ surfaces with heterogenous sites. This phenomenon is not rare, as similar findings were already reported in literature [39,41,42,43] and could be explained by understanding the surface chemistry composition of MWNTs_ox_ entrapped on the surface of PDMS/MWNT_ox_ sponge. After the oxidation process, different chemical moieties with different abundances and non-uniform distribution are introduced on MWNT surfaces. This could cause differences in the energy level of the active sites available on the PDMS/MWNT_ox_ sponge surface, thus affecting the adsorption capacity. Active sites with higher energy levels tend to form heterolayer phenolic compound coverage with robust support from strong chemical bonding, while active sites with lower energy levels will induce monolayer coverage, due to electrostatic forces [39,41,42,43].

The obtained results were compared with different types of sorbent used for removal of phenolic compounds (Table 1). As evident the here-studied adsorbent evidenced a good adsorption capacity for both the adsorbates with higher and lower affinity with the PDMS/MWNT_ox_ sponge. The highest values reported in literature were achieved with coconut shells and MWNT_ox_ powder [46]. Their maximum adsorption capacity, *q*_max_, is almost one order of magnitude higher compared to that obtained in the present study, but it should be considered that herein, the adsorption capacities are calculated per gram of sponge and not per gram of MWNT_ox_. With the consideration that the removal of phenolic compounds is attributed solely to the MWNTs_ox_ present in the sponge (as demonstrated by adsorption kinetic studies, Figure 4), the *q*_max_ calculated per gram of MWNT is 374.6 mg/g for 4-NP, and 216.87 mg/g for phenol, thus reaching higher values than in [45,46]. Moreover, the here-proposed materials have the additional advantages of being user-friendly and easy to recover after adsorption processes.

More in details, in a real application, the use of other proposed materials requires post-treatment processes (e.g., filtration, centrifugation) to remove the adsorbent from the aqueous solution after the uptake process. In the current study, MWNTs_ox_ are entrapped in a macroporous polymeric sponge, easy to manipulate and separate, thus offering significant advantages in terms of costs and usability with respect to other approaches.

### 3.4. Adsorption Kinetic Studies

To elucidate the adsorption process mechanism of PDMS/MWNT_ox_ sponge, three kinetic models were used to test the experimental data, i.e., the pseudo-first order equation, the pseudo-second order equation, and the intraparticle diffusion equation. All the studies were performed in a solution containing 0.18 mM of 4-nitrophenol or phenol in the presence of the same amount of adsorbent phase.

### 3.5. The Pseudo-First Order and Pseudo-Second Order Kinetic Model

**Figure 8:**
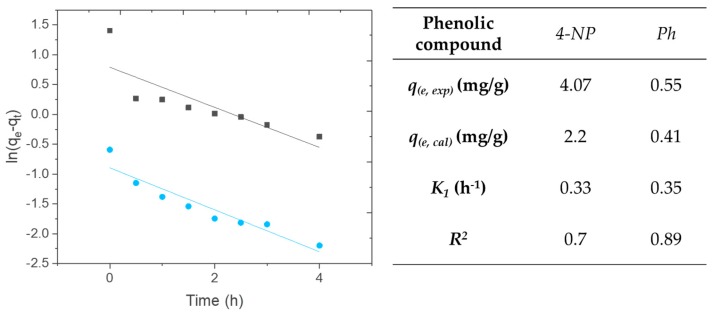
Application of pseudo-first order adsorption model for the adsorption of 4-NP (black square) and phenol (blue circles) onto PDMS/MWNT_ox_ sponges. The table reports the calculated values from Equation (8).

The pseudo-first order kinetic model is more suitable for low concentrations of solute. The pseudo-first order equation is

(8) $$\ln(q_{e}-q_{t})=\ln q_{e}-k_{1}t$$

where *q_e_* and *q_t_* refer to the amount of phenolic compounds adsorbed (mg/g) at equilibrium and at any time, *t* (h), respectively, and *k*_1_ (h^−1^) is the equilibrium rate constant of pseudo-first order sorption. The plot of ln(*q_e_* − *q_t_*) against t should give a straight line with slope −*K*_1_ and intercept ln*q_e_*, as can be seen in Figure 8. The calculated parameters are reported in the table. The correlation coefficient for 4-NP and Ph were 0.7 and 0.89, respectively, and the relative calculated value of *q_e_* (*q*_(*e*,*cal*)_) were in both cases smaller than experimental ones (*q*_(*e*,*exp*)_). The fitting results indicated that the experimental data did not agree well with this model.

**Figure 9:**
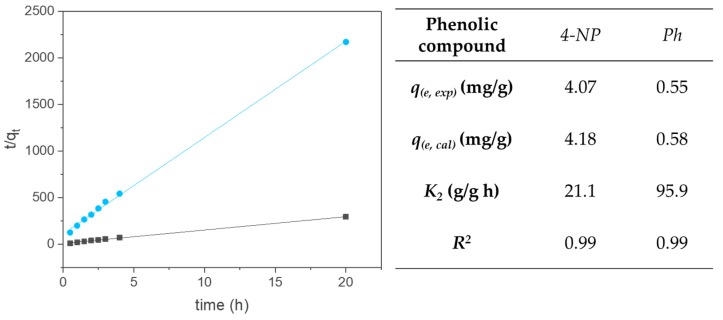
Application of pseudo-second order adsorption model for the adsorption of 4-NP (black square) and phenol (blue circles) onto PDMS/MWNT_ox_ sponges. The table reports the calculated values from Equation (9).

The pseudo-second order equation is dependent on the amount of the solute adsorbed on the surface of adsorbent, and the amount adsorbed at equilibrium, and is expressed in the form

(9) $$\frac{t}{q_{t}}=\frac{1}{k_{2}q_{e}^{2}}+\frac{t}{q_{e}}$$

where *k*_2_ is the rate constant of pseudo-second order equation, *q_t_*, *q_e_* and *t* have the same meaning as that in pseudo-first order equation. From the slope and the intercept of the plot of *t*/*q_t_* versus *t* (Figure 9), the rate constant (*k*_2_) and the equilibrium adsorption capacity (*q_e_*) can be obtained. As observable in the table reported in Figure 8, the correlation coefficients (*R*^2^) were higher than 0.99 for both the tested phenolic compounds, and the calculated *q_e_* values are close to the experimental ones. These findings suggest that pseudo-second order model fits, remarkably well, the experimental data, making it suitable to explain the kinetics for the adsorption of phenolic compounds onto the PDMS/MWNT_ox_ sponge. In more detail, it suggests that the chemical sorption in the adsorption process [39] is mainly due to π–π interactions, as previously demonstrated with isotherm studies.

### 3.6. The Intraparticles Diffusion Model

Although the pseudo-first order and the pseudo-second order models can explain the adsorption process, none of them could identify the diffusion mechanisms, and so then the intraparticle diffusion model was also used to analyze and elucidate the diffusion mechanism. The intraparticle diffusion equation can be written in the following form:

(10) $$q_{t}=k_{p}t^{1/2}+C$$

where *k_p_* is the rate constant of intraparticle diffusion model, *C* is a constant for any experiment (mg/g), and *q_t_* has the same meaning as that in Equation (9). By plotting *q_t_* versus *t*^1/2^ curves, two linear ranges were obtained for both the tested phenols, thus indicating the occurrence of at least two diffusion steps during the adsorption process.

**Figure 10:**
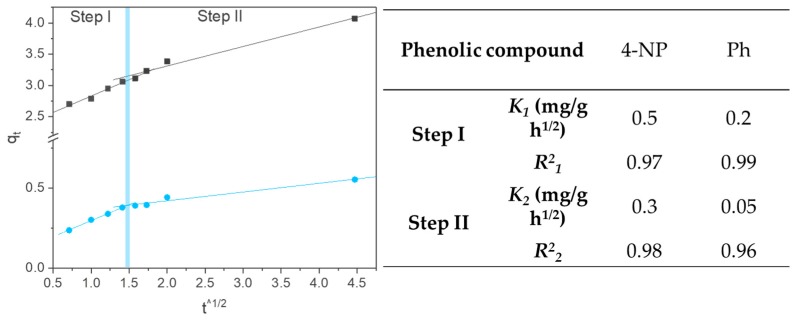
Application of intraparticle diffusion model for the adsorption of 4-NP (black square) and phenol (blue circles) onto PDMS/MWNT_ox_ sponges. The table reports the calculated values from Equation (10).

As shown in Figure 10, from the slope of each linear curve, *k_p_* values can be obtained. From the extrapolation of the first step in the curve to the time axis, the intercept C could be obtained [47]. As seen in table reported in Figure 10, most of the correlation coefficients are higher than 0.97 for both the phenolic compounds tested, which suggested that the adsorption of phenolic compounds by the PDMS/MWNT_ox_ sponge fit this model well. The slope of the linear portion indicated the rate of diffusion; a larger *k* corresponds to a faster diffusion process. Hence, *k*_1_ > *k*_2_ (with *k*_1_ and *k*_2_ representing *k_p_* values for step I and II, respectively) indicated the free path available for diffusion became smaller, thus leading to the reduction rate in the diffusion process. In more detail, it could be hypothesized that the external surface adsorption or diffusion in macropores occurred at the first step, until the outer part reached saturation. After this process, the phenolic compounds entered into less accessible pores, or in small portions between two adjacent carbon nanotubes, so the diffusion resistance increased, and the diffusion rate decreased. Consequently, the second step was the final equilibrium step for which the adsorbed molecules move slowly from larger pores to smaller pores, and in regions between adjacent MWNTs, causing a slow adsorption rate [48]. The interpretation of this phenomena was already reported in literature [47,49,50].

### 3.7. Reusability Studies

Reusability studies were performed to check the recyclability of the PDMS/MWNT_ox_ spongeous materials, and better elucidate the mechanism of adsorption. After a first adsorption process, three different sponges were washed in three different solutions: water, 5% acetic acid (pH = 2.4), and alkaline water (pH = 12), and used for another adsorption process. The experiments were performed for both 4-NP and Ph. In all cases, the washing solutions were heated at 70 °C, it is well known in fact that π–π interactions are weaker at this temperature [51]. After acetic acid treatment, the PDMS/MWNT_ox_ sponges demonstrated a recovery of the original uptake of ~96% for 4-NP and ~98% for Ph, suggesting that after this procedure, most of the adsorption sites are recovered due to the desorption of phenolic compounds. The washing procedure with warm water evidenced a recovery of lower than 8% for both the tested phenolic compounds, suggesting strong interactions between these species and the PDMS/MWNT_ox_ sponge are present. A recovery of ~63% for 4-NP and 52% for Ph was obtained after washing at pH = 12, probably because at this pH, most of the oxygen-containing moieties on MWNT_ox_ surface and phenolic compounds are in the deprotonated form, favoring the desorption of part of the phenols through electrostatic repulsion.

Moreover, as already reported in literature, the higher recovery of binding sites in the presence of organic acids can be confirmed, as the adsorption is held by the sorbent through chemisorption process for both the tested phenolic compounds [39].

### 3.8. Removal of Phenolic Compound from Olive Mill Wastewater

The efficacy of the proposed PDMS/MWNT_ox_ sponge was evaluated by performing an adsorption test in a complex matrix, such as oil mill wastewater (OMW). Material corresponding to 1.2% in weight of adsorbent was tested at ambient temperature, under stirring and 24 h of contact time with OMW, in which the amount of phenolic compound was equal to 1250.9 ppm, calculated with the Folin-Ciocalteu assay. The results showed that with this amount of PDMS/MWNT_ox_ sponge, we obtained an elimination of 32.3% of total phenolic compounds, suggesting the possibility to use the material as an adsorbent phase for phenolic compounds in complex matrices. A proper decontamination of OMW could be obtained by the increase of the adsorbent phase or the number of adsorption cycles.

## 4. Conclusions

In conclusion, the present work describes the formation of a nanocomposite sponge made of PDMS and oxidized MWNTs for the removal of phenolic compounds from water. The MWNT loading was performed during the fabrication of the sponge without the use of solvent and/or complex procedures. The oxidation of MWNTs was necessary to increase sponges’ affinity toward phenolic compounds, and was performed directly on the as-produced foam, simplifying the post-treatment washing procedure and speeding up the whole process. The as-prepared materials showed high adsorption capacity with different affinity towards different phenolic compounds. As demonstrated, this is basically due to the different π–π electron donor-acceptor interactions between adsorbate and adsorbent, depending on the different type and number of substituents on aromatic rings of phenolic compounds. The formation of an heterogenous monolayer during the adsorption process onto PDMS/MWNT_ox_ sponge surface was demonstrated by Langmuir and Freundlich isotherm studies. The pseudo-second order kinetic model was found to represent the adsorption kinetic process with higher correlation coefficients than the pseudo-first order kinetic model, confirming the chemical interaction between the phenolic compounds and the spongeous material. In addition, two different steps for adsorption procedure were identified with the intraparticle diffusion model, probably depending on the presence of pores with different sizes, and on the low accessibility of binding sites between adjacent MWNTs.

The entrapment of MWNTs_ox_ adsorbent in a porous polymeric matrix represents a significant advantage with respect to other approaches presented for the adsorption of phenolic compounds. Usually, post-treatment processes, such as filtration or centrifugation, are required to remove the (nano)adsorbent phase after the adsorption process, increasing time and costs of the treatment. These problems are avoided with the here-proposed approach. Moreover, the here-prepared sponges evidenced good adsorption capacity, suggesting that the synthetic pathway did not significantly affect adsorption capacity of MWNTs. The proposed method of preparation could be also extended to other (nano)adsorbents to facilitate the handling and safe disposal of the nanomaterials.

To further demonstrate the adsorption capacity of the materials, a complex matrix, such as OMW sample, was treated with the nanocomposite to remove phenolic compounds, demonstrating their effective removal, and suggesting their possible use for the treatment of wastewater. Moreover, the surface material could be regenerated towards an acidic treatment, thus decreasing the costs of production in wastewater treatment application.

## Figures and Tables

**Table 1:** Langmuir and Freundlich constants for phenolic compound adsorption from various absorbents reported in literature.

Adsorbent	Adsorbate	*q*_max_ (mg/g)	*K_L_* (L/g)	*K_F_*	*n*	References
PDMS/MWNTs_ox_	4-Nitrophenol Phenol	25 14.29	0.03 0.03	363.83 27.98	3.29 1.77	This work
Resin AP-246	Phenol	0.071	0.584	0.112	0.35	[42]
Activated coal	Phenol	1.84	0.065	0.79	0.79	[42]
Hydroxyapatite	Phenol			0.37	1.66	[44]
Resin OC-1074	Phenol	0.043	0.445	0.0053	0.16	[42]
Coconut shell	Phenol	205.84	3.91	37.11	3.66	[45]
MWNT–COOH	4-nitrophenol	256.41	0.05	23.15	1.34	[46]

## Supplementary Materials

The following are available online at http://www.mdpi.com/2079-4991/8/5/334/s1.
