# Supplementary material for: An Innovative Porous Nanocomposite Material for the Removal of Phenolic Compounds from Aqueous Solutions
Source: Nanomaterials (Basel). 2018 May 16;8(5):334. doi: 10.3390/nano8050334 (PMC5977348; doi:10.3390/nano8050334)
Supplement: Supplementary file 1 [file nanomaterials-08-00334-s001.pdf]

Innovative porous nanocomposite material for the removal of phenolic compounds from aqueous solution.

Antonio Turco, Anna Grazia Monteduro, Elisabetta Mazzotta, Giuseppe Maruccio, Cosimino Malitesta

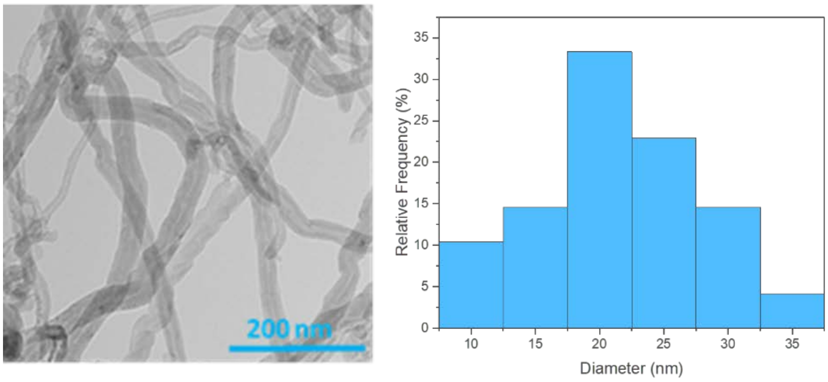

Figura S 1 Representative TEM image of MWNTs (left) and their size distribution (right).

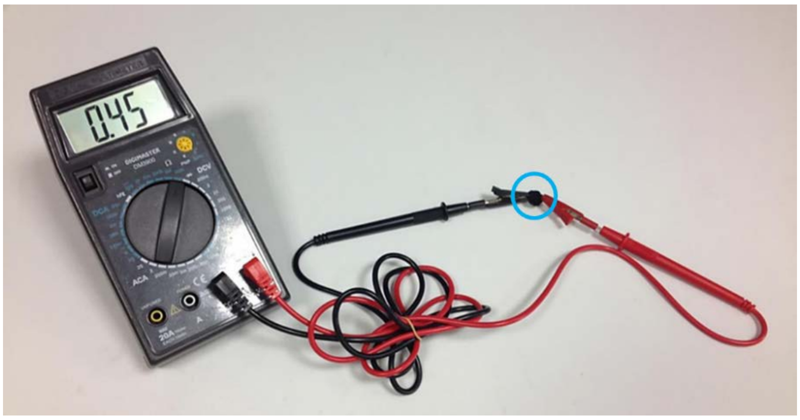

Figura S 2 Conductivity behavior of PDMS-MWNTs$_{ox}$ sponge (in blue circle) evidenced by conducting the material with a multimeter

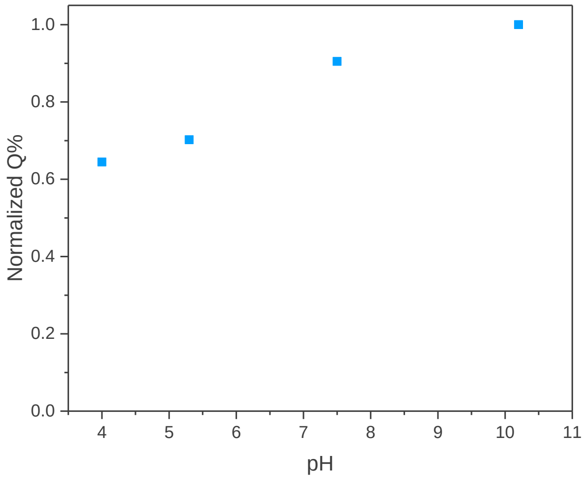

Figura S 3 Effect of pH on adsorption of 4-Nitrophenol by PDMS-MWNTs$_{ox}$ sponge

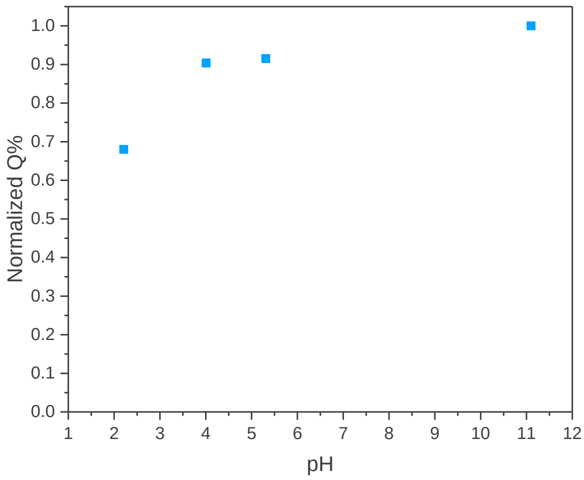

Figura S 4 Effect of pH on adsorption of Phenol by PDMS-MWNTs$_{ox}$ sponge
